# Comparative Transcriptional Analysis of Clinically Relevant Heat Stress Response in *Clostridium difficile* Strain 630

**DOI:** 10.1371/journal.pone.0042410

**Published:** 2012-07-30

**Authors:** Nigel G. Ternan, Shailesh Jain, Malay Srivastava, Geoff McMullan

**Affiliations:** Northern Ireland Centre for Food and Health (NICHE), School of Biomedical Sciences, University of Ulster, Coleraine, Co. Londonderry, North Ireland, United Kingdom; The Roslin Institute, University of Edinburgh, United Kingdom

## Abstract

*Clostridium difficile* is considered to be one of the most important causes of health care-associated infections worldwide. In order to understand more fully the adaptive response of the organism to stressful conditions, we examined transcriptional changes resulting from a clinically relevant heat stress (41°C versus 37°C) in *C. difficile* strain 630 and identified 341 differentially expressed genes encompassing multiple cellular functional categories. While the transcriptome was relatively resilient to the applied heat stress, we noted upregulation of classical heat shock genes including the *groEL* and *dnaK* operons in addition to other stress-responsive genes. Interestingly, the flagellin gene (*fliC*) was downregulated, yet genes encoding the cell-wall associated flagellar components were upregulated suggesting that while motility may be reduced, adherence – to mucus or epithelial cells – could be enhanced during infection. We also observed that a number of phage associated genes were downregulated, as were genes associated with the conjugative transposon Tn5397 including a group II intron, thus highlighting a potential decrease in retromobility during heat stress. These data suggest that maintenance of lysogeny and genome wide stabilisation of mobile elements could be a global response to heat stress in this pathogen.

## Introduction

*Clostridium difficile* a Gram-positive anaerobic spore-forming bacterium, is now considered to be one of the most important causes of health care-associated infections [1] and is said to be the most frequent cause of infectious bacterial diarrhoea worldwide [2]. *C. difficile* infection (CDI), which is primarily associated with the use of broad spectrum antibiotics to treat other underlying conditions, has three main stages: initial alteration of the indigenous colonic microflora by broad-spectrum antibiotics; germination of *C. difficile* spores, with cellular multiplication unhindered by colonisation resistance due to disruption of the indigenous microflora, and finally, the release of the two main virulence factors, toxins A and B [3]. These result in the classical symptom associated with CDI: mild to severe watery diarrhoea [4], [5]. Most patients also present with abdominal pain and cramping, in association with leukocytosis and low grade fever which may involve a temperature as high as 40.6°C [6]. Numerous reports show that both the incidence and severity of CDI has risen significantly in the last decade [1], [7], [8]. The fundamental reason for this increase is the alarming emergence of hypervirulent strains of *C. difficile* (e.g., ribotype 027/NAP1, responsible for 34% of reported UK CDI cases) [9], [10] which possess an expanded repertoire of antibiotic resistance elements, produce greater quantities of toxins, and thus increase the severity of disease with higher mortality rates and an increased probability of relapse following clinical treatment [11], [12].

The publication by Sebaihia et al [13] of the complete genome sequence of *C. difficile* strain 630, followed in recent years by sequencing and subsequent comparative genetic and phylogenomic analyses of over 30 *C. difficile* genomes [2], [14]–[16] has led to the identification of a relatively large 3.4 Mb core genome for the species. *C. difficile* as a whole is a genetically diverse species, however – recent publications show that *C. difficile* genomes can contain a vast spectrum of genes primarily involved in antimicrobial resistance, virulence, host interaction, production of surface structures and other metabolic capabilities allowing survival within the challenging gut environment. A highly effective and virulent pathogen has thus evolved relatively quickly [15], [17].

Comparatively little is known about the adaptive response of *C. difficile* to stresses encountered during CDI. Emerson et al [18] began to address this knowledge gap by analysing the transcriptional responses of *C. difficile* strain 630 to a variety of antimicrobial and environmental stresses, and the recent work of Janvilisri et al [19] and Scaria et al [20], using a cell culture model and an *in vivo* CDI model, respectively, has built upon this, identifying a number of significantly regulated genes, operons and pathways unique to, or common between, different stresses.

We recently generated a reference proteome defining the response of *C. difficile* strain 630 to a clinically relevant heat stress (41°C v 37°C) using 2D-LC-MS/MS and isobaric labelling [21]. We identified a distinct functional proteomic profile comprising some 12% of the theoretical proteome. Proteomic investigations are limited by the proteome coverage obtained, however, as this depends largely upon the instrumentation used. As part of our systems biology approach to defining the heat stress response of *C. difficile*, we utilised microarray analysis to obtain a global perspective and we now report a transcriptomic analysis of the same mild, prolonged, clinically relevant heat stress in *C. difficile* strain 630.

## Materials and Methods

### Bacterial Cell Culture

*Clostridium difficile* strain 630 was routinely maintained on BHI agar or grown in BHI broth (Oxoid) at 37°C in a MACS MG500 Anaerobic workstation fitted with an airlock (Don Whitley Scientific, UK). Heat stress was induced in broth cultures in the early exponential phase of growth using a water bath set at 41°C and cells were harvested in biological triplicates at late log phase (D_650_ = 1.1) of anaerobic growth as described by Jain et al [21].

### Total RNA Isolation

RNA was extracted from aliquots of 4×10^8^ cells from both control and heat-stressed triplicate cultures of *C. difficile* strain 630 using a Qiagen RNEasy mini kit. The Qiagen protocol was modified to include a mechanical lysis step – cells in TE buffer with proteinase K and lysozyme were added to a Lysing Matrix A tube (MP Biomedicals) and treated in a Fastprep FP120 machine (MP Biomedical) at speed 5.5 for 30 s to break open the cells. Following both on-column and in-solution DNAse digestions, and a final on column cleanup, RNA samples were confirmed free of contaminating genomic DNA by performing PCR with *tpi* primers [22]. RNA Samples were stored at −70°C until required for microarray experiments or for qRT-PCR.

### Template Labelling and Microarray Hybridisations

Microarray experimentation was out-sourced to Oxford Gene Technology (OGT; Begbroke Science Park, Oxford, UK). RNA samples were sent on dry ice to OGT where the quality and integrity of the 16S and 23S ribosomal RNA (rRNA) subunits was verified by using the 2100 Bioanalyzer system (Agilent Technologies). For all six RNA samples, an RNA integrity number of >9.6 was obtained, with A260/280 values of >2.0, and 23S∶16S rRNA ratios of ≥1.4. Using Ambion's MessageAmp™ II-Bacteria RNA Amplification Kit, the template mRNA samples were: (a) polyadenylated; (b) the mRNA samples with a stable poly(A) tail were reverse-transcribed into first strand cDNA using an oligo(dT)-primer bearing a T7 promoter; (c) the cDNA samples were then converted into double-stranded DNA (dsDNA); (d) dsDNA was then used as a template for *in vitro* transcription with T7 RNA polymerase to generate antisense RNA (aRNA); (e) aRNA was then finally labelled with fluorescent dyes (Cy3 and Cy5) to create labelled probes for hybridisation. In this investigation, a dye-swap (i.e. control samples labelled with Cy3 and heat-stress samples labelled with Cy5 and vice versa) was performed in order to generate technical replicates and to compensate for any potential bias introduced as a result of inherent discrepancies in Cy dye incorporation [17], [23]. Prior to hybridisation, labelled aRNA was purified using Qiagen's RNeasy® MinElute Cleanup Kit as per the manufacturer's instructions. The labelled probes were then hybridised to a *C. difficile* strain 630 array (BUGS CD630 gene expression array plus Plasmid pCD630, 8×15k array, v2.01) comprising 3,776 genes using the Gene Expression Hybridisation Kit (Agilent Technologies) as described in the manufacturer's protocol.

### Microarray Data Analysis

The hybridised arrays were subsequently scanned at 532 nm and 635 nm, corresponding to Cy3 and Cy5 excitation maxima, using an Agilent C Microarray Scanner equipped with the extended dynamic range (XDR) software for improved resolution. The data was then extracted from raw microarray image files and the probe signals were subsequently quantified using Agilent's Feature Extraction Software version 10.5.1.1. Upon normalisation by the locally weighted scatter plot smoothing (LOWESS) algorithm, the data was imported to GeneSpring GX version 11.0 (Agilent Technologies) where the minimum fluorescence intensity was set to 1. The mean normalised log_2_ fluorescence ratios and standard errors of mean were then calculated across all probes for an individual gene. To test for statistical differences between the heat-stress and control condition, a paired T test against zero change log ratio, with a 5% confidence level, was applied in conjunction with the Benjamini-Hochberg False-Discovery Rate (FDR) multiple testing correction, essentially as per Janvilisri et al [19]. All subsequent analyses of the filtered datasets were performed using Microsoft Excel. Of the 3776 genes present on the *C. difficile* strain 630 array, 3769 were available for comparative studies. Initially the microarray data were filtered to exclude genes that had a *p* value of ≥0.05. In previous work [21], we used a 1.5 fold cutoff for biologically significant perturbations. Whilst we could have employed a cutoff value of 1.2 fold (the value suggested by the paragon algorithm in the Protein Pilot software), at that time we adopted a conservative approach in order to increase the robustness of our conclusions and a cutoff value of 1.5 (i.e. log ratios between −0.585 and 0.585) was chosen. Thus, for consistency in the present work, we have considered as differentially expressed (DE), only those genes with *p*<0.05 that also changed by 1.5 fold. The data discussed herein has been deposited in NCBI's Gene Expression Omnibus [24] and is accessible through GEO Series accession number GSE37442 (http://www.ncbi.nlm.nih.gov/geo/query/acc.cgi?acc=GSE37442).

### Reverse Transcription and qPCR

Differential gene expression data was validated using qRT-PCR as previously described by Jain et al [21]. Briefly, 100 ng of total RNA isolated from the control and heat-stressed *C. difficile* strain 630 cells was reverse-transcribed to cDNA using SuperScript® II Reverse Transcriptase (Invitrogen) and the reverse primers of the individual genes targeted. The triose phosphate isomerase gene, *tpi* (CD3172), and 16S rRNA were used as reference genes [22] and 2.5 µl cDNA was used as template in 10 µl q-PCR reactions (LightCycler 2.0 Carousel-Based System) using the LightCycler FastStart DNA MasterPLUS SYBR Green I kit (Roche diagnostics GmbH, Mannheim, Germany). q-PCR was performed in technical triplicates to enable accurate measurement of C_q_ [25] and the LightCycler® amplification protocol comprised an initial denaturation at 95°C for 10 min, followed by 45 cycles of 95°C for 10 s, annealing and fluorescence acquisition at 55°C for 10 s and elongation at 72°C for 20 s. The temperature transition rate was 20°C/s for each step. For each qPCR reaction, the specificity of the amplification was assessed by performing a melting curve analysis, and PCR products were confirmed as the expected size by gel electrophoresis and sequencing.

## Results and Discussion

### Transcript profile of heat stressed C. difficile strain 630 cells

**Figure 1:**
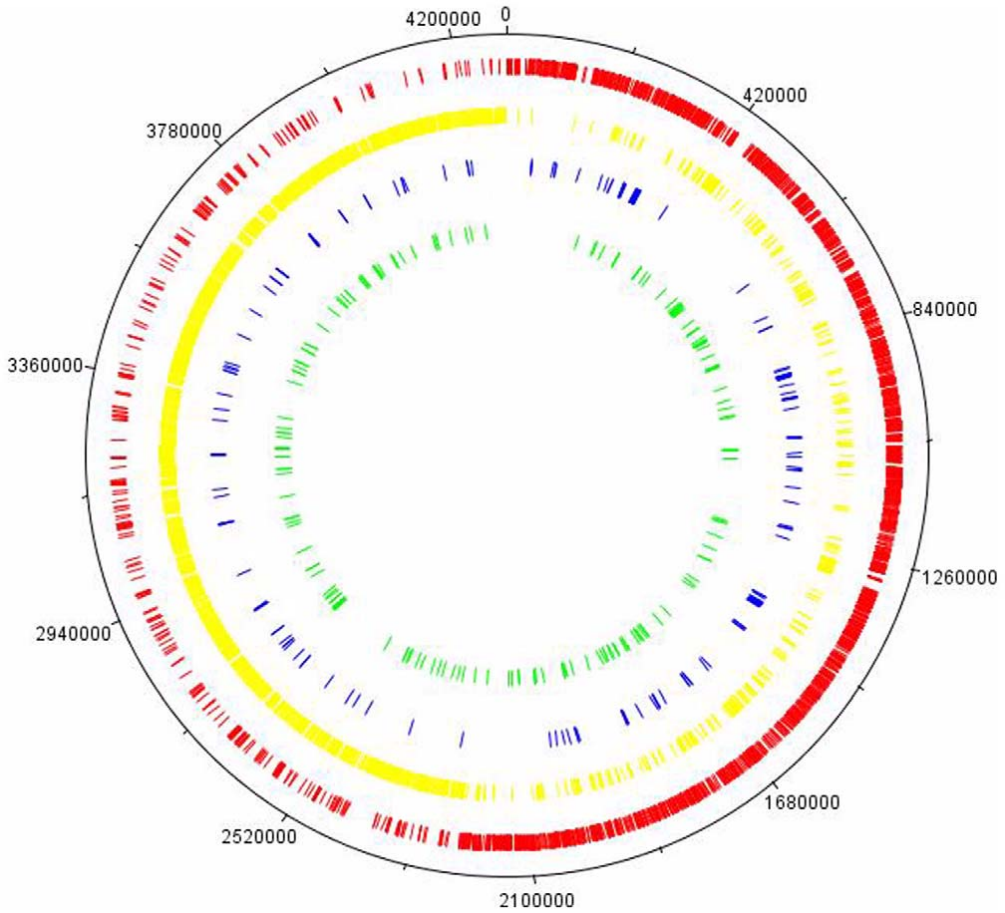
Projection of differentially expressed genes on the *Clostridium difficile* 630 genome. Those genes showing at least 1.5-fold change with *p*<0.05 were selected. From outside to inside: Ring 1, molecular clock of *C. difficile* strain 630 genome; Ring 2 (red), coding DNA sequences of the forward strand of the genome; Ring 3 (yellow), coding DNA sequences(CDS) of the opposite strand of the genome; Ring 4 (blue), genes upregulated at 41°C; Ring 5 (green), genes downregulated at 41°C.

Transcriptomic analysis of *C. difficile* strain 630 cells exposed to a mild but prolonged and clinically relevant heat stress (41°C versus 37°C) revealed a total of 427 transcripts with p<0.05. Of these, 341 transcripts, constituting 9% of the array total and spanning the entire genome (Figure 1), were differentially expressed (DE) at >1.5 fold (162 up, 179 down). The complete list of genes, highlighting significant expressional differences, with functional categorisation and mapped to our previously published iTRAQ proteomics data, is given in Table S1. Our iTRAQ proteomics dataset contained a validated list of 447 proteins, of which 49 were DE at 41°C (30 up, 19 down) as described in Jain et al [21]. Some 40 DE gene products were common to both the iTRAQ proteomics and the microarray datasets: we noted agreement between the datasets for 6 genes (*dnaK*, *grpE groEL*, *groES*, *fliC*, *prdF*). A “direction of perturbation” agreement, with the caveat that either the 1.5-fold cutoff for biological significance or the *p* value for statistical validity was not satisfied in the iTRAQ proteomics data, was identified for a further 10 genes. A total of 13 gene products that were not DE in the iTRAQ proteomics data were also unchanged in the array dataset. Thus, 53 gene products were common to both proteomics and array data, with 19 (35%) showing agreement, while 30 (57%) did not. In addition, it should be noted that 4 gene products (CD2924:phage protein, CD2020:*clpB*, CD1357:petidyl prolyl isomerase, and CD2532:aminotransferase) that were identified as DE at 41°C in the iTRAQ proteomics dataset were excluded from the comparison as their microarray *p* values were >0.05 (Table S2). Overall however, the microarray proved to be a much more sensitive high throughput technique, with the capacity to reliably identify a wider range of DE gene products and yielding quantitative data for an additional 301 DE genes that were not identified in the iTRAQ experiment. The transcriptomic analysis was characterised by higher sensitivity than the iTRAQ proteomics experiment, which is unsurprising as LC/MS/MS dynamic range is somewhat limited by the sensitivity of the mass spectrometer used in addition to a number of other workflow factors including protein extraction, labelling efficiency, chromatographic fractionation and the data dependent nature of MS/MS analyses.

**Figure 2:**
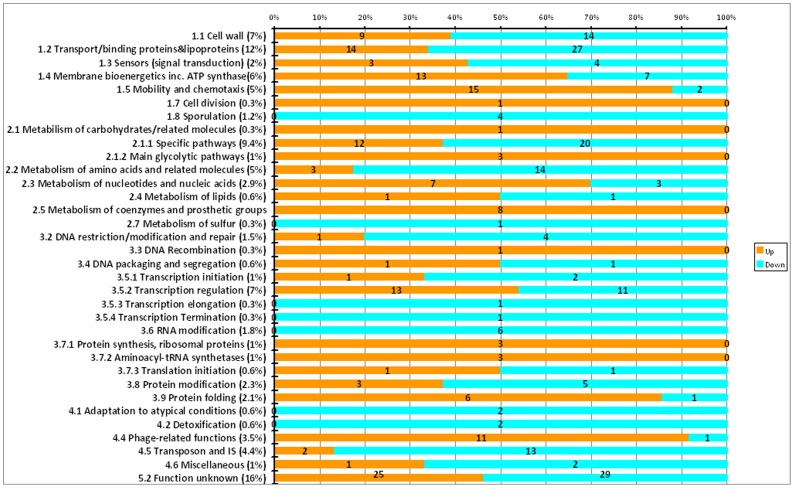
Functional category annotation of differentially expressed genes (>1.5 fold, *p*<0.05) in the *Clostridium difficile* strain 630 heat stress transcriptome. Key: Orange – up regulated; blue – down regulated.

Multiple functional categories [26], [27] were identified within the subset of DE genes (Figure 2), including genes involved in the cell wall/envelope (23), transport (41), membrane bioenergetics (20), transcriptional regulators (24) and genes involved in specific pathways (32). In addition, some 54 genes categorised as encoding proteins “similar to unknown proteins” were differentially expressed at 41°C. Within the subset of DE genes, the largest numbers of upregulated transcripts at 41°C were in the categories of similar to unknown (25/54), mobility and chemotaxis (15/17), transport (14/41 genes), membrane bioenergetics (13/20 genes), transcriptional regulation (13/24 genes), specific pathways (12/32) and phage related functions (11/12). With respect to transcripts downregulated at 41°C, categories were as follows: similar to unknown (29/54), transport (27/41), specific pathways (20/32), metabolism of amino acids (14/17), cell wall functions/related (14/23), transposons and IS (13/15) and transcriptional regulation (11/24) (Figure 2).

### Validation of microarray data using qRT-PCR

**Figure 3:**
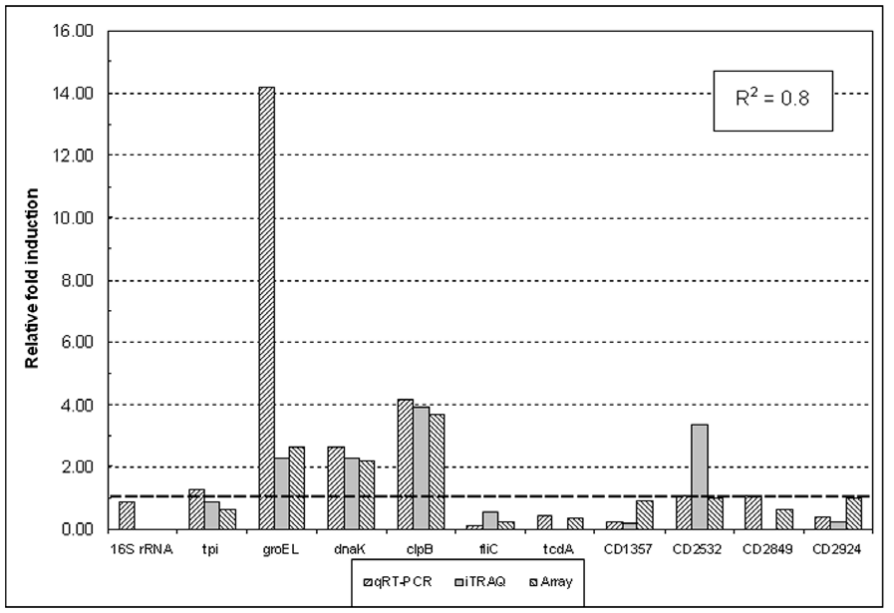
Comparison of qRT-PCR, iTRAQ proteomics and microarray data for selected *Clostridium difficile* strain 630 genes. For each individual gene, expressional fold-change values (up-hatched columns) are shown relative to the 37°C control. Corresponding iTRAQ fold-changes (gray columns) are included for comparison with microarray data (down-hatched columns) and show good correlation between the three data sets. 16S rRNA, *tpi*, and CD2849, whose expression did not change by more than 1.5-fold, were used as reference genes.

Our previous work showed good correlation between iTRAQ proteomics data and subsequent qRT-PCR analysis [21] as did the current work. Differences in gene expression by microarray analysis appeared to be underestimated, however the direction of the perturbations at 41°C was the same and linear regression analysis of the log_2_ values yielded an R^2^ correlation coefficient of 0.80 (Figure 3), comparable to that described by other researchers [19], [20].

### Biological interactions

Using the STRING (Search Tool for the Retrieval of Interacting Genes/Proteins at http://string-db.org/) database [28] to predict functional interactions within the cell for DE genes allowed visualisation of functionally related genes that were not necessarily encoded contiguously within the genome (Figure S1 & Figure S2). Genes involved in heat stress response, motility, phage functions, *de-novo* thiamine biosynthesis, amino acid metabolism, and several transcriptional regulators (CD1293, CD1292; CD2307, CD2308; CD3139, CD3140) associated with hypothetical proteins were identified. We observed upregulation of genes associated with conversion of pyruvate to acetyl Co-A (*acoA*, *acoB*, *acoC*, CD0036–CD0038), and of *plfD*, formate acetyltransferase (CD0759), which converts acetyl-CoA and formate to CoA and pyruvate, during metabolism of propanoate and butanoate. CD1054 and CD1056, encoding butyryl-CoA dehydrogenase (*bcd2*) and an electron transfer flavoprotein alpha-subunit (*etfA2*), two enzymes of the butanoate metabolic pathway were also upregulated. The phosphotransferase (PTS) sugar transport systems were by and large unperturbed by the applied heat stress, with the exception of PTS genes for transport of fructose (CD3015), cellobiose (CD3049) and mannose (CD0285–CD0287). Components of the sorbitol specific PTS system (CD0765–CD0767) were upregulated at 41°C, as were a number of genes involved in introduction of phosphorylated sorbitol and glucose to the fructose and mannose metabolism pathways (CD0768, CD3064, CD2324). These perturbations could be a means of adaptation to different carbon sources for energy generation, or for biosyntheses of amino sugars or recycling of nucleotides.

A *de novo* purine biosynthesis operon, encoded by CD0218–CD0225 showed upregulation of the first three genes (*purE*, *purC*, *purF*), although no change could be established in transcripts of the remainder of the operon (*p*>0.05). It has been proposed that *de novo* purine biosynthesis is necessary for virulence of *Candida albicans* in a mouse model [29] and for virulence of *Burkholderia pseudomallei* [30], thus our results indicate that additional factors contributing to *C. difficile* virulence will be modulated under heat stress. StringDB analysis also revealed the relationship between *de novo* purine biosynthesis and methylenetetrahydrofolate biosynthesis, where upregulation of CD0719, CD0720 and CD0722 was observed at 41°C. Genes encoding components of the carbon monoxide dehydrogenase/acetyl-CoA synthase complex (CD0723–CD0728), which generates the central metabolite, acetyl CoA, were upregulated as were genes encoding components of *de novo* thiamine biosynthesis (CD1702, CD1702A, [*thiC*, *thiS*] and CD1599, CD1600, CD1601 [*thiD*, *thiK*, *thiE1*]), suggesting an increased requirement for biosynthesis of various vitamins, including thiamine and folates, under heat stress conditions. While gut microbes are known to contribute to synthesis of vitamins in the human GI tract [31], this apparent increase in thiamine synthesis could also be explained by an increased requirement for TPP type riboswitch ligands for precise control of gene expression under variable conditions [32].

StringDB analysis of down regulated genes showed well defined gene clusters associated with the conjugative transposon Tn5397, the PTS sugar transport systems as mentioned above and the glycine reductase complex (CD2347–CD2357) which interacts with certain thioredoxins (CD2355, CD2356; CD1690, CD1691; CD2117). A number of less well defined clusters of genes including sensor kinases and response regulators (CD1465, CD3266, CD0643, CD3267 CD3320) of unknown function, as well as other genes involved in regulatory and nucleic acid processing/degradation functions were down regulated at 41°C. These included a regulatory endoribonuclease (CD3187, *tdcF*), ribonuclease PH (CD3308), an excinuclease ABC subunit B (CD3412, *uvrB*), endonuclease III and RNA methylase (CD0565, CD0566), and a putative ATP-dependent RNA helicase (CD0761). These data suggest that overall there is a significant change in the fate of mRNA transcripts under heat stress at 41°C, possibly leading to longer half lives due to decreased post-transcriptional processing by these enzymes.

StringDB predicted a functional relation ship between a putative ABC transporter solute binding protein (CD3525, 2.45 fold down) and a “putative bifunctional protein (CD2849, 1.5 fold down) containing two domains – an N-terminal phosphonatase like domain, and a C-terminal 2-aminoethylphosphonate (2AEP) transaminase like domain. Whether this fusion protein – which has most likely arisen due to genomic rearrangement – retains any functionality towards 2AEP or phosphonoacetaldehyde is unknown. Expression of the degradative genes generally occurs only in the presence of 2AEP [33], [34], yet our data show that these genes are transcribed under “normal” growth conditions (∼15 mM Pi), in the absence of 2AEP. Their precise mode of regulation, therefore, and specific biological functions, are thus open to further investigation.

### Heat Stress response

Heat shock proteins (Hsps) are evolutionarily conserved, suggesting similar roles in the diverse range of life in which they are found and perhaps the best characterised are those Hsps with chaperone functions or proteolytic activities [35], [36]. In the model Gram positive organism *Bacillus subtilis*, heat shock genes/proteins are categorised into one of at least six classes [37]–[39]. In the current investigation, we noted a classical response at 41°C by members of the HrcA regulon (Class I heat shock genes). Thus induction of *groES* (CD0193), *groEL* (CD0194), *dnaJ* (CD2460), *dnaK* (CD2461), *grpE* (CD2462) and *hrcA* (2463) was observed at 41°C, an observation consistent with our work [21] and that of others [18]. Of the general stress response (Class II) genes which are regulated by σ^B^, we noted, as did Emerson et al [18], that expression of the *sigB-rsbV-rsb*W operon (CD0009–CD0011) was unchanged. This is not entirely unexpected, as the response of σ^B^ to general stresses, including heat stress, is of a transient nature [40]. Helmann et al [35] showed that, in *B. subtilus*, expression of σ^B^ was maximally induced after a mere 3 min at a higher temperature and greatly reduced following 20 min incubation, whereas our experiment took place over 3 h, by which time it might be expected that transcription of these genes would have returned to a basal level. The CTSR regulon (Class III heat shock genes) includes the Clp (caseinolytic protein) proteases, which were unchanged in our investigation, as was the expression of *htpG*, the single member of the class IV heat shock genes. However, we have previously identified an increase in abundance of Clp1 (CD3305), ClpB (CD2020) and ClpC (CD0026) and HtpG (CD0273) in our iTRAQ proteomics dataset [21]: these ClassIII heat shock genes are however excluded from the current microarray dataset of DE genes due to their p values being >0.05, although raw data suggests upregulation. Whilst there is generally a correlation between proteomic and transcriptomic data, in many cases the correlation is only weakly positive [41], [42] suggesting that other effects are involved in translational regulation. For example, analysis of *Lactobacillus rhamnosus* GG showed that for a transcriptome of 316 DE genes and a proteome of 42 DE proteins, only 14 gene products showed a correlation between transcript and protein [43]. Indeed we observed, as have others [44], that transcriptome and proteome data for *C. difficile* strain 630 showed only a qualitative agreement regarding expressional differences, and that between studies absolute values differed greatly [18]. Numerous explanations may be hypothesised for these discrepancies. Factors including transcription efficiency [42], protein stability/stabilisation, or the presence of small regulatory RNAs [32] that have the potential to stabilise or promote degradation of transcripts, could all play a role and it is clear that the correlation between mRNA and protein abundance is not clear cut.

The two members of the CssRS regulon that comprise the known Class V heat shock genes, namely *htrA* and *htrB* [38], [39] play a role in sensing the accumulation of misfolded proteins at the membrane cell wall-interface [45], [46]. *C. difficile* 630 appears to contain homologues of *htrA* (CD3284 and CD1633) which were unaffected in our experiment. BlastP analysis of the *C. difficile* 630 genome using *B. subtilus* or *E. coli htrB* sequences also revealed lesser similarity to both CD3284 and CD1633. Furthermore, expression of the two component regulatory system encoded by CD1013 and CD1014 (homologues of *cssR*/*cssS*, respectively) was unchanged, and thus the exact components of a *C. difficile* class V heat shock regulon, responsive to prolonged heat stress, remain unknown. Of the genes belonging to the Class VI Heat shock category including *lon* (CD3301), *clpX* (CD3304), and *ftsH2* (CD3559), no expressional change in response to the applied heat tress was observed, save for *ftsZ* (CD2646) which was upregulated at 41°C, as was an upstream tricistronic operon postulated to have a role in cell division (CD2647–CD2649).

Our observations from both proteomic and transcriptomic investigations point to the fact that in *C. difficile*, as in *B. subtilus*, there exist different mechanisms for temperature sensing associated with each category of heat shock gene. Schumann [47] hypothesised the existence of different thermal induction mechanisms, with DNA, mRNA, or proteins (e.g. transcription factors, chaperones and proteases) acting as heat sensors. For one mechanism, as long as the heat stimulus is present, gene expression is activated. Thus for the *groEL* and *dnaK* operons, temperature upshift results in the appearance of denatured proteins, following which the sensor chaperones and proteases are titrated by these “non-native” polypeptides, leading to stable, increased heat shock gene expression. The alternative sensor mechanism results in transient gene expression, regardless of continued heat stress, such as that described for genes controlled by *sigB* [35], [39].

### Flagella, Motility and Chemotaxis

**Figure 4:**
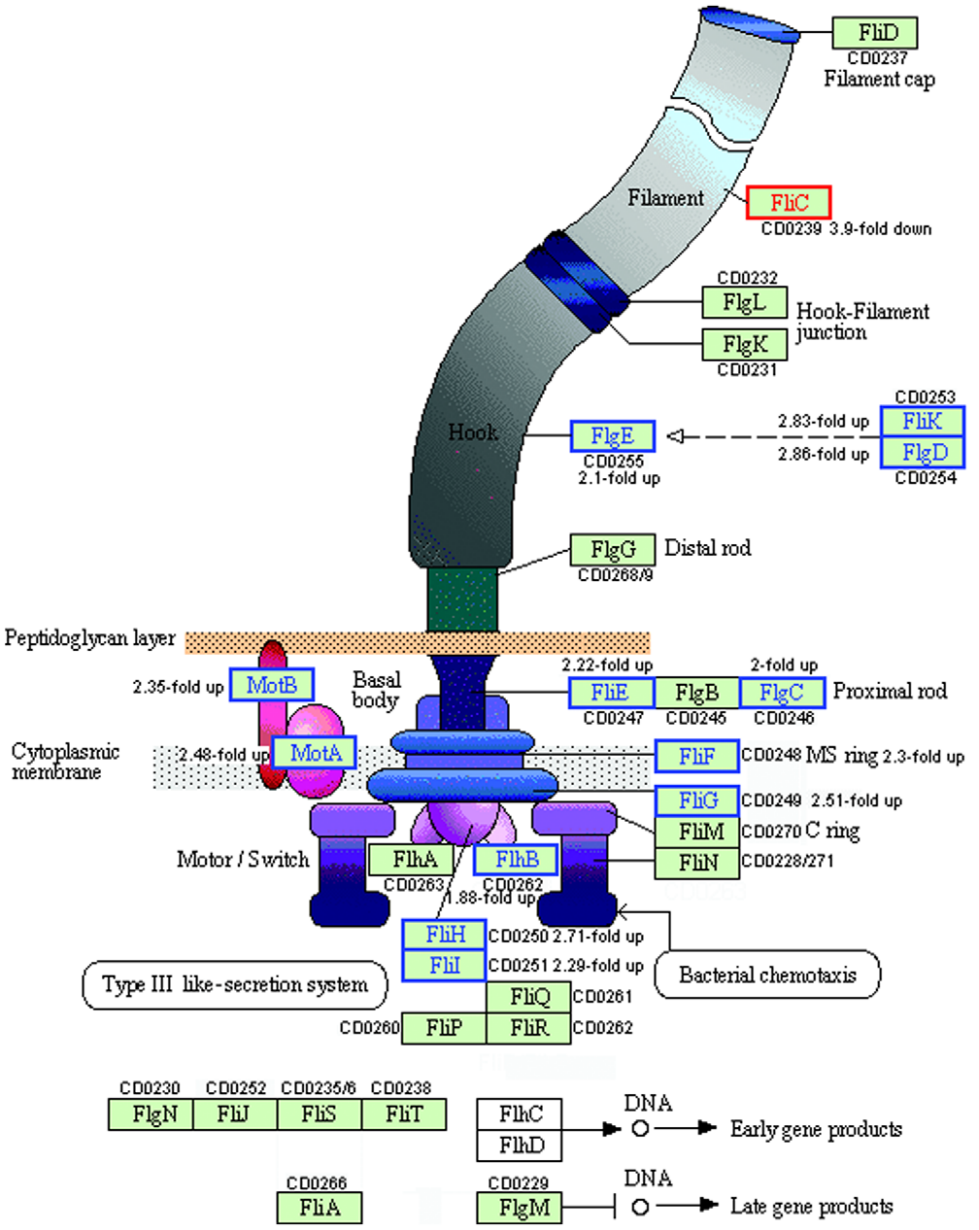
The flagellar motor assembly in *Clostridium difficile* strain 630. Graphic is adapted from Kyoto Encyclopedia of Genes and Genomes (http://www.genome.jp/kegg/). Several genes involved in the flagellar assembly showed altered expression levels in response to heat-stress. Additional genes found in *C. difficile* (*FlgN*, *fliA*, *FliT*) were added following BLAST searching. There appear to be no homologues of the master regulator, *FlhD*/*C* in *C. difficile* strain 630. Key: Red box, down regulated >1.5 fold; blue box, up regulated >1.5 fold, black box, no statistically significant change.

In *C. difficile* strain 630, the genes encoding the protein machinery of the flagella are arranged in two separate loci. The F1 locus spans CD0226–CD0240, while the F2 locus spans CD0245–CD0271. These loci are separated by a four gene region known as the interflagellar locus, F2, containing genes involved in flagellar glycan biosynthesis one of which, CD0240, was necessary for proper flagellar assembly and thus motility [48], [49]. The assembly of the flagella is a highly ordered process, involving the hierarchal control and expression of numerous genes. Flagellar biogenesis is accepted to be in the order of the basal body, the hook, and finally, the filament (Figure 4). Initially, the interaction of *flhD* and *flhC* gene products is responsible for activation of class II flagellar genes; this FlhDC complex is therefore the master regulator of flagellar gene expression. Class II flagellar genes include the basal body, hook and the alternative sigma factor, *FliA*, along with its cognate anti-sigma factor, *flgM*. Class III genes include flagellin (*fliC*) itself and other late-stage genes requiring *fliA* for their transcription. In our investigation, upregulation of the genes encoding components of the basal body, motor, proximal rod, flagellar hook and molecular ruler, encoded by CD0253 and CD0254, was observed at 41°C (Figure 4). Interestingly a significant down-regulation of the gene encoding flagellin (*fliC*, CD0239) was observed whilst the filament cap gene (*fliD*, CD0237) was unchanged. This differential expression of *fliC* and *fliD* is in agreement with our previously published proteomics data [21] and furthermore supports the recent findings of Dingle et al [50] who investigated the importance of the flagellum in *C. difficile* pathogenesis using a hamster model. Using Clostron technology to inactivate *fliC* or *fliD*, the authors surprisingly concluded that the flagella, and motility, did not contribute to adherence to epithelial cells *in vitro*. Indeed, they argued that flagella are either not necessary for virulence, or that repression of motility could be a pathogenic mechanism. Thus it would appear from our ’omic datasets that *C. difficile* cells exposed to a clinically relevant heat stress may increase their ability to adhere to epithelial cells and possibly their virulence. However virulence is a multi factorial phenomenon and therefore these observations should be taken in the light of the work of Karlsson et al [51] who observed that increasing the growth temperature above 37°C in fact decreased expression of toxins A and B in *Clostridium difficile* VPI10463. Expression of the *tcdA* gene (CD0663) was 3-fold downregulated at 41°C in the microarray dataset and in addition, we have also observed a decrease in TcdA protein expression via gelC-MS driven proteomics (Ternan et al, unpublished), lending weight to the argument that virulence is reduced as temperatures move away from 37°C [52]. *TcdB* (CD0660) was not DE in the current analysis. The data for toxin production and motility are therefore similar despite our groups using different strains of *C. difficile*: *C. difficile* VPI10643 is a high toxin producer in serogroup G, while *C. difficile* strain 630 is most closely related to serogroup C and produces much lower toxin levels [53], [54]. However, it is clear that similarities in the regulation of toxin production and motility exist at a fundamental level: it may be hypothesised that adherence increases as toxin production falls during the latter stages of CDI when a patient is likely to have pyrexia due to the infection [6]. Interestingly, a number of known virulence factors, including *cwp20* (CD1469), *cwp5* (CD2786), and CD3567 – encoding a cell wall hydrolase – were down regulated in our microarray dataset, adding further evidence that virulence of *C. difficile* is ‘set’ at 37°C.

### Group II intron

**Figure 5:**
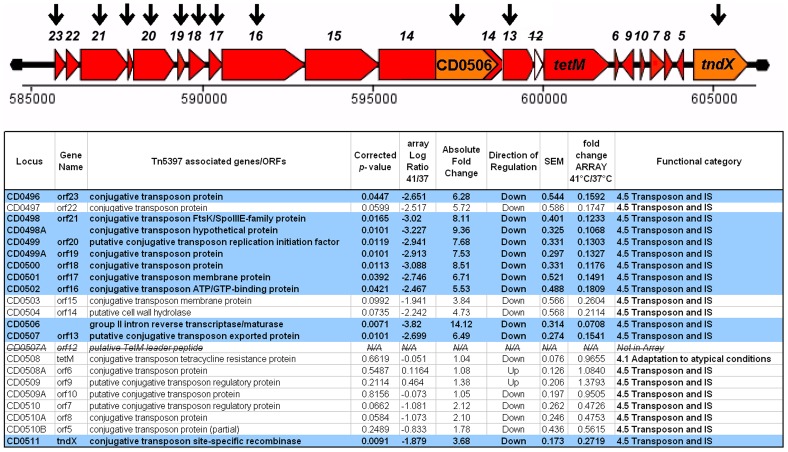
Genomic context of Transposon Tn5397 in the genome of *Clostridium difficile* strain 630. Key: Arrows represent direction of changes in gene expression; genes lacking an arrow are unchanged. Tn5397 ORF numbers are shown above the genes. CD0506: group II intron. *tetM*: tetracycline resistance determinant.

The most down regulated gene in our experiment was CD0506 (14 fold down regulated at 41°C, *p* = 0.007), which encodes the group II intron that is recognised as ORF12 of the ∼20 kb conjugative transposon, Tn5397 [55], [56]. Tn5397 is responsible for the plasmid-independent transfer of tetracycline resistance not only between *C. difficile* isolates but also to strains of *B. subtilis*, *Staphylococcus aureus*, *Enterococcus faecalis* and *Streptococcus acidominimus* [57]. We noted considerable downregulation of almost the entire Tn5397 region in response to heat stress at 41°C. Interestingly, however, the tetracycline resistance determinant, *tetM* (CD0508), and the six Tn5397 ORFs downstream of *tetM*, were unchanged (Figure 5). The *tetM* data from our microarray experiment is thus in agreement with our proteomics dataset [21].

Precisely how group II introns function and mobilise in a cell, or indeed how they respond to environmental or nutritional insults to their hosts, is not fully characterised. However, Group II introns have been proposed to interact with a range of cellular metabolites associated with stress, including cAMP, ppGpp and polyphosphate levels [58], [59]. It would appear that in *C. difficile* 630, heat stress could result in a significant decrease in retromobility. The cellular implications of this observation remain unknown. We hypothesise that while the ability of *C. difficile* to survive tetracycline treatment remains unchanged, the ability of the resistance determinant to be transferred, or even lost from the genome, appears to be significantly decreased. A number of phage related proteins, including an integrase (CD3153–CD3156), were upregulated at 41°C, suggesting that maintenance of lysogeny, and genome wide stabilisation of mobile elements, could be a global response to heat stress.

### Conclusion

The DE genes identified in this investigation considerably extend our understanding of global transcriptional regulation in response to clinically relevant heat stress in *C. difficile* strain 630. It is evident from the literature however that bacterial transcriptomes and the mechanisms by which they are controlled are unexpectedly complex [60], [61], suggesting that an integration of transcriptomic and proteomic data is required to obtain a comprehensive molecular characterisation of a biological system [44]. Our proteomic [21] and transcriptional profiles of *C. difficile* strain 630 under heat stress should inform the selection of targets for development of antimicrobial therapies, functional genomics studies and modelling of cellular processes. Thus the research community is provided with a platform which will help to facilitate a deeper understanding of this pathogen via the in depth investigation of individual genes and operons that are key to the organism's survival under adverse conditions.

## Supporting Information

Figure S1 **StringDB representation of interactions between up regulated genes in** ***Clostridium difficile*** **strain 630 under heat stress.**
(TIF)

Figure S2 **StringDB representation of interactions between down regulated genes in** ***Clostridium difficile*** **strain 630 under heat stress.**
(TIF)

Table S1 **List of *Clostridium difficile*** **strain 630 genes with p<0.05 showing differential expression and highlighting significant expressional differences, with functional categorisation and mapped to previously published (Jain et al, 2011) iTRAQ proteomics data.** Genes up-regulated by a fold change of 1.5 or more are highlighted in orange, and those down-regulated by a fold change of 1.5 or more are highlighted in blue.
(XLS)

Table S2 **Entire microarray expression data for heat stressed** ***Clostridium difficile*** **strain 630.** Genes up-regulated by a fold change of 1.5 or more are highlighted in orange, and those down-regulated by a fold change of 1.5 or more are highlighted in blue. The remainder of the genes were not statistically significantly differentially expressed.
(XLS)
