# Supplementary figures and images for: Comparative Transcriptional Analysis of Clinically Relevant Heat Stress Response in Clostridium difficile Strain 630
Source: PLoS One. 2012 Jul 30;7(7):e42410. doi: 10.1371/journal.pone.0042410 (PMC3408451; doi:10.1371/journal.pone.0042410)

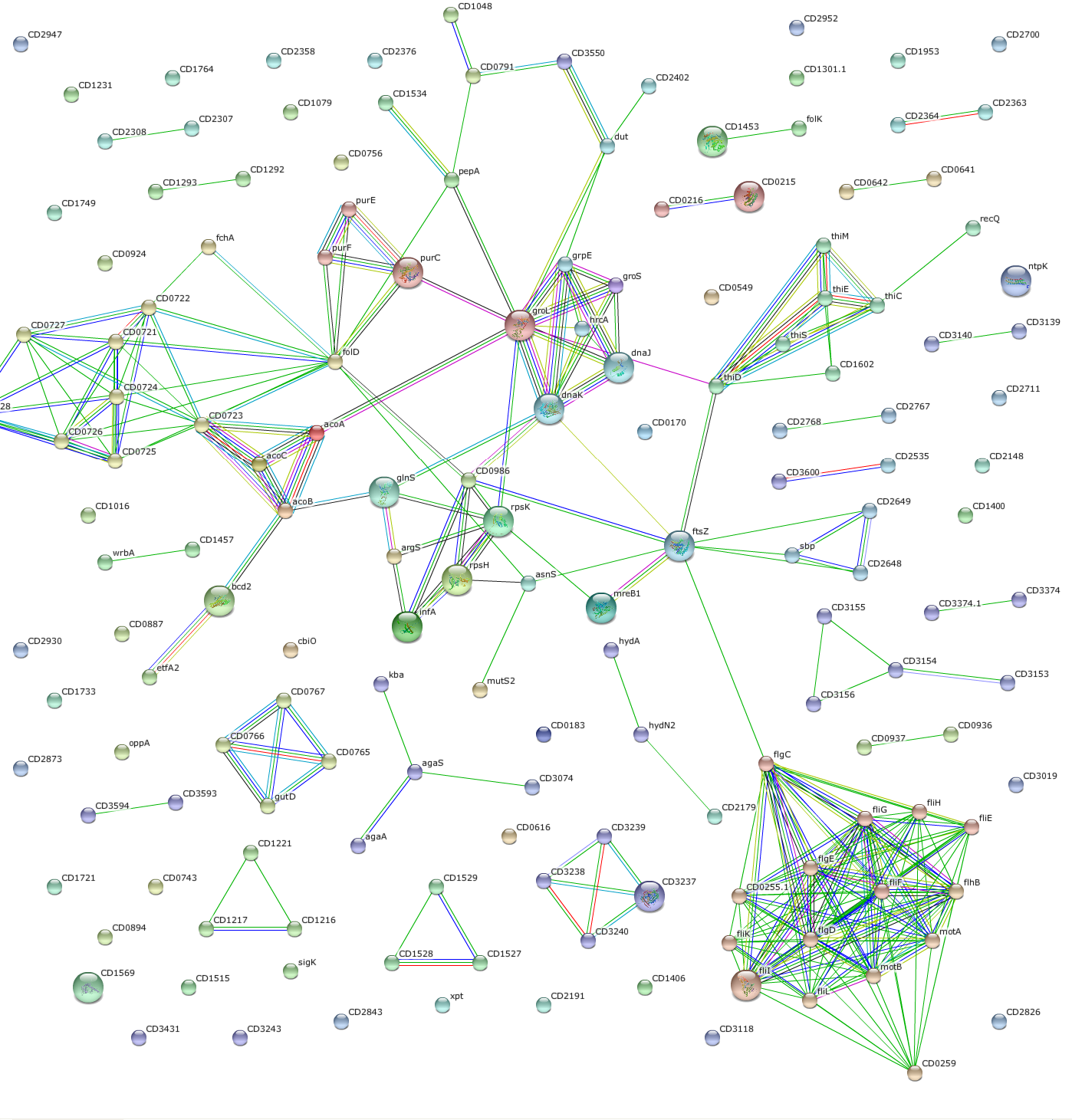

Supplement: Figure S1 — StringDB representation of interactions between up regulated genes in Clostridium difficile strain 630 under heat stress. (TIF) [file pone.0042410.s001.tif]

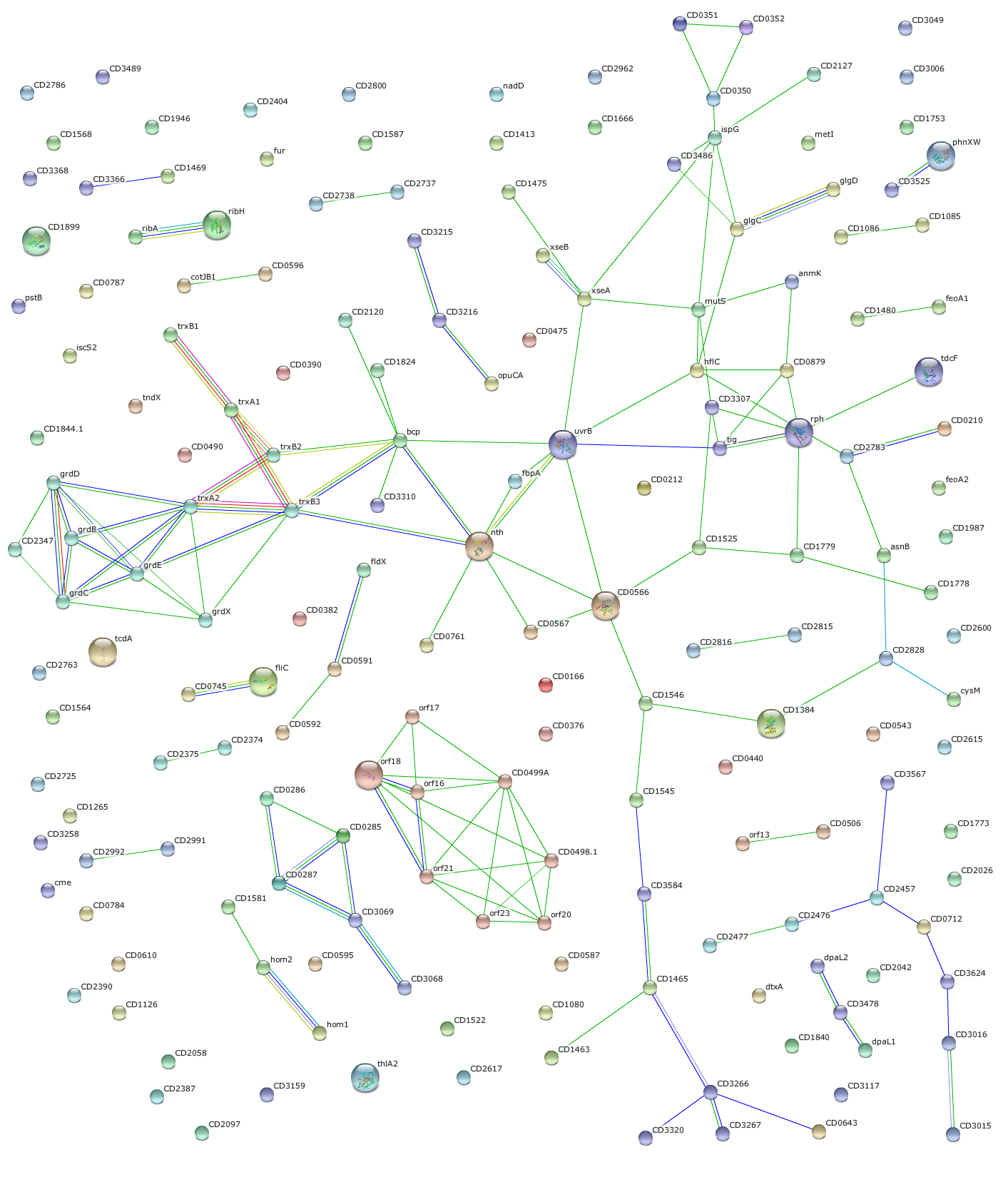

Supplement: Figure S2 — StringDB representation of interactions between down regulated genes in Clostridium difficile strain 630 under heat stress. (TIF) [file pone.0042410.s002.tif]
